# Hard ticks in Burmese amber with Australasian affinities

**DOI:** 10.1017/S0031182022001585

**Published:** 2023-02

**Authors:** Lidia Chitimia-Dobler, Jason A. Dunlop, Timo Pfeffer, Felix Würzinger, Stephan Handschuh, Ben J. Mans

**Affiliations:** 1 Bundeswehr Institute of Microbiology, Neuherbergstrasse 11, 80937 Munich, Germany; 2 Museum für Naturkunde, Leibniz Institute for Evolution and Biodiversity Science, Invalidenstrasse 43, D-10115 Berlin, Germany; 3 Keyence Deutschland GmbH, Siemensstrasse 1, D-63263 Neu-Isenburg, Germany; 4 VetCore Facility for Research/Imaging Unit, University of Veterinary Medicine Vienna, Veterinärplatz 1, A-1210 Vienna, Austria; 5 Epidemiology, Parasites and Vectors, Agricultural Research Council-Onderstepoort Veterinary Research, Onderstepoort, South Africa; 6 Department of Life and Consumer Sciences, University of South Africa, Pretoria, South Africa

**Keywords:** *Archaeocroton*, Australia, *Bothriocroton*, Cretaceous, fossil, Ixodida, Metastriata, Myanmar, New Zealand

## Abstract

Three examples of metastriate hard ticks (Ixodida: Ixodidae) with apparent affinities to modern Australasian genera are described from the mid-Cretaceous (ca. 100 Ma) Burmese amber of Myanmar. Two nymphs of *Bothriocroton muelleri* sp. nov. represent the oldest (and only) fossil record of this genus, living members of which are restricted to Australia and predominantly feed on monitor lizards, snakes and echidnas. A female of *Archaeocroton kaufmani* sp. nov. shares its basis capitulum shape with the tuatara tick *Archaeocroton sphenodonti* (Dumbleton, 1943), the only extant member of this genus and an endemic species for New Zealand. The presence of 2 Australasian genera in Burmese amber is consistent with a previous record of an *Ixodes* Latreille, 1795 tick from this deposit which resembles Australian members of this genus. They further support an emerging hypothesis that fauna of the amber forest, which may have been on an island at the time of deposition, was at least partly Gondwanan in origin. A revised evolutionary tree for Ixodida is presented compiling data from several new Burmese amber ticks described in the last few years.

## Graphical abstract

**Figure:**
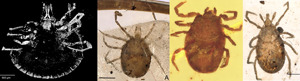

## Introduction

Ticks (Arachnida: Parasitiformes: Ixodida) are haematophagous ectoparasites found on several vertebrate hosts. For an overview of their biology and economic significance as disease vectors, see Sonenshine and Roe (2013). About 900 living species are known (Guglielmone *et al*., 2010), divided across 3 families and 20 extant genera. Molecular data (Mans *et al*., 2012, 2019) suggest that the group may have originated during the Permian, but fossils are only known from the mid-Cretaceous or younger. For recent summaries, see, e.g. de la Fuente (2003), Dunlop *et al*. (2016), Chitimia-Dobler *et al*. (2017, 2018, 2022) and Peñalver *et al*. (2017). An extinct family and genus (Deinocrotonidae: *Deinocroton* Peñalver *et al*., 2017), possibly related to the extant Nuttalliellidae, was described from the Cretaceous (ca. 100 Ma) Burmese amber outcropping in Myanmar. A second extinct family, Khimairidae, has since been recognized (Chitimia-Dobler *et al*., 2022), and the same amber deposit yields the oldest hard ticks (Ixodidae). These Burmese amber fossils currently comprise examples of the extant genera *Ixodes* Latreille, 1795 (Chitimia-Dobler *et al*., 2022), *Amblyomma* CL Koch, 1844 (Chitimia-Dobler *et al*., 2017) and *Haemaphysalis* (Allocereae) CL Koch, 1844 (Chitimia-Dobler *et al*., 2018), as well as 2 extinct genera, *Cornupalpatum* Poinar and Brown, 2003, and *Compluriscutula* Poinar and Buckley, 2008, whose affinities probably lie close to *Amblyomma*.

Burmese amber thus yields an increasingly diverse and interesting tick fauna, both in terms of extinct taxa and the oldest records of several living groups. Here, 3 new ticks from amber are described which are of particular biogeographical significance with respect to an ongoing debate (e.g. Poinar, 2018) about whether Burmese amber hosts a fauna with Laurasian or Gondwanan affinities. Two well-preserved nymphs represent the first, and so far only, fossils referable to the extant Australian genus *Bothriocroton* Keirans, King and Sharrad, 1994, a group which is today found on monitor lizards, snakes and the short-beaked echidna among the monotremes. The third amber fossil is a female referred to *Archaeocroton* Barker and Burger, 2018 which resembles the extant tuatara tick (cf. Godfrey *et al*., 2008) from New Zealand. The new material draws 2 more modern tick genera back into the mid-Cretaceous and is consistent with the hypotheses that the so-called metastriate ticks (i.e. all hard ticks excluding *Ixodes*) radiated during the Early Cretaceous (Mans *et al*., 2012), or the Jurassic (Mans *et al*., 2019). In this context, the provisional evolutionary tree presented in Chitimia-Dobler *et al*. (2017, Fig. 3) is revised and updated to include both the new fossils from the present study and other published records in the recent literature.

## Materials and methods

The 2 nymphs assigned to *Bothriocroton* come from the collection of Mr Patrick Müller and bear the original inventory numbers BUB1544 and BUB1573. The female assigned to *Archaeocroton* originates from the collections of the first author and has been deposited in the Museum für Naturkunde, Berlin (accession number MB.A. 4452). Burmese amber mostly comes from deposits in the Hukawng valley of northern Myanmar and has been dated to the mid-Cretaceous (earliest Cenomanian), or about 98.79 ± 0.62 Ma (Shi *et al*., 2012). A subsequent study by Smith and Ross (2018) based on bivalve borings in the resin suggests that there has been only minimal reworking of the inclusions, and a Cenomanian age of about 100 Ma for the fossils may be appropriate. Further details about the history of discovery and the geological setting can be found in Grimaldi *et al*. (2002) and Ross *et al*. (2010); see also Selden and Ren (2017) for a recent review focused on the arachnids. An updated list of Burmese amber inclusions can be found in Ross (2021).

For photography, a Keyence VHX-6000 digital microscope (Keyence Itasca, IL, USA) with a tiltable stand and a combination of incident and transmitted light for focus stacking was used (with 100× to 1000× magnification). Image stacks were combined using software Helicon Focus 6.7.1 and polarized light was used to obtain more details. For computed tomography (*μ*-CT), the *Archaeocroton* sample was mounted on a specimen holder and scanned using a Zeiss Xradia MicroXCT-400 (Carl Zeiss X-Ray Microscopy, Pleasanton, CA, USA) at 40 kVp tube voltage. The tomography was acquired using the 4X detector assembly. The reconstructed image volume was processed and visualized by volume rendering using 3D software package Amira 6.4. Drawings were prepared with a *camera lucida* attachment on a Leica M205C stereomicroscope (Leica Microsystems, Wetzlar, Germany), again using a combination of incident and transmitted light where appropriate.

The *Archaeocroton* fossil is a female, which shows significant damage to 1 side of its body. All legs on the left side are broken as well as a little part of the idiosoma. The fact that part of the legs (tarsi) are broken and have an unusual position and all broken segments are very dry are signs of possible tick damage by the host during grooming and not during the polishing process of the amber piece.

## Results

### Systematic palaeontology for the *Bothriocroton*

Class Arachnida Lamarck, 1801

Order Parasitiformes Reuter, 1909

Suborder Ixodida Leach, 1815

Family Ixodidae Murray, 1877

*Bothriocroton* Keirans, King and Sharrad, 1994

*Bothriocroton muelleri* Chitimia-Dobler, Mans and Dunlop sp. nov.

*Etymology*: In honour of Mr Patrick Müller (Kashöfen), who has made some extraordinary fossil ticks in Burmese amber available for study.

*Material*: Holotype BUB1544 and paratype BUB1573 (coll. P. Müller). Burmese amber, Myanmar, Late Cretaceous (Cenomanian).

*Diagnosis*: Body oval, scutum subtriangular with few large punctuations, the second article of palps at least twice as long as the third article, 11 festoons, eyes absent, spiracle plates comma-shaped extruding noticeably from lateral body margins, coxae I–IV with external spurs, tarsus I strongly humped and tarsus IV humped.

**Fig. 1.**
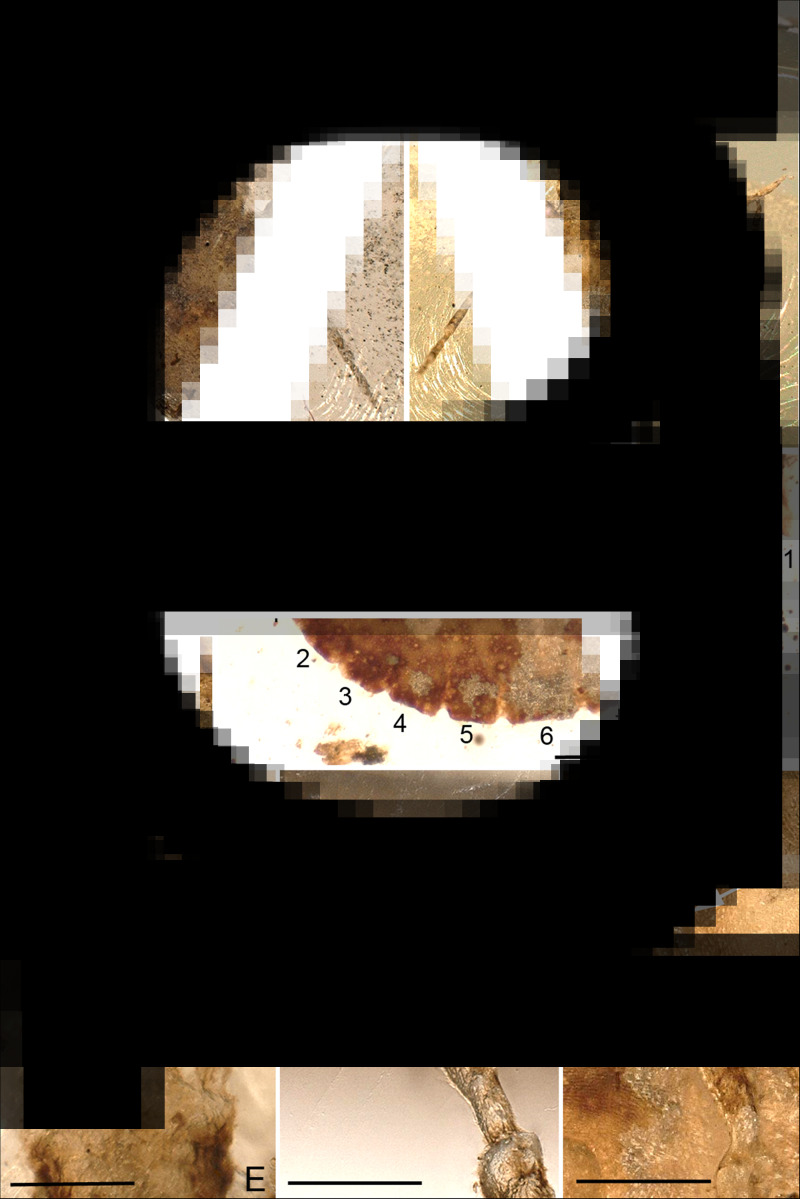
Holotype of *Bothriocroton muelleri* sp. nov., P. Müller collection no. BUB1544, from Late Cretaceous (ca. 100 Ma) Burmese amber from Myanmar. (A) Dorsal overview. (B) Ventral overview. (C) Coxal spurs (arrowed). (D) Posterior idiosoma showing Y-shaped anal groove, extruded spiracle plates (arrowed) and 11 festoons (numbered). (E) Capitulum, including palps with long second article and hypostome with 2/2 dentition. (F) Leg I tarsus with prominent distal hump (arrowed) plus 6 additional lobes. (G) Leg IV tarsus with small hump (arrowed). Scale bars equal 1 mm (A, B), 250 *μ*m (C), 500 *μ*m (D) and 200 *μ*m (E–G).

*Description of the holotype*: BUB1544 is an unengorged nymph (Fig. 1); dorsal surface partially obscured because of scratches in the matrix.

**Fig. 2.**
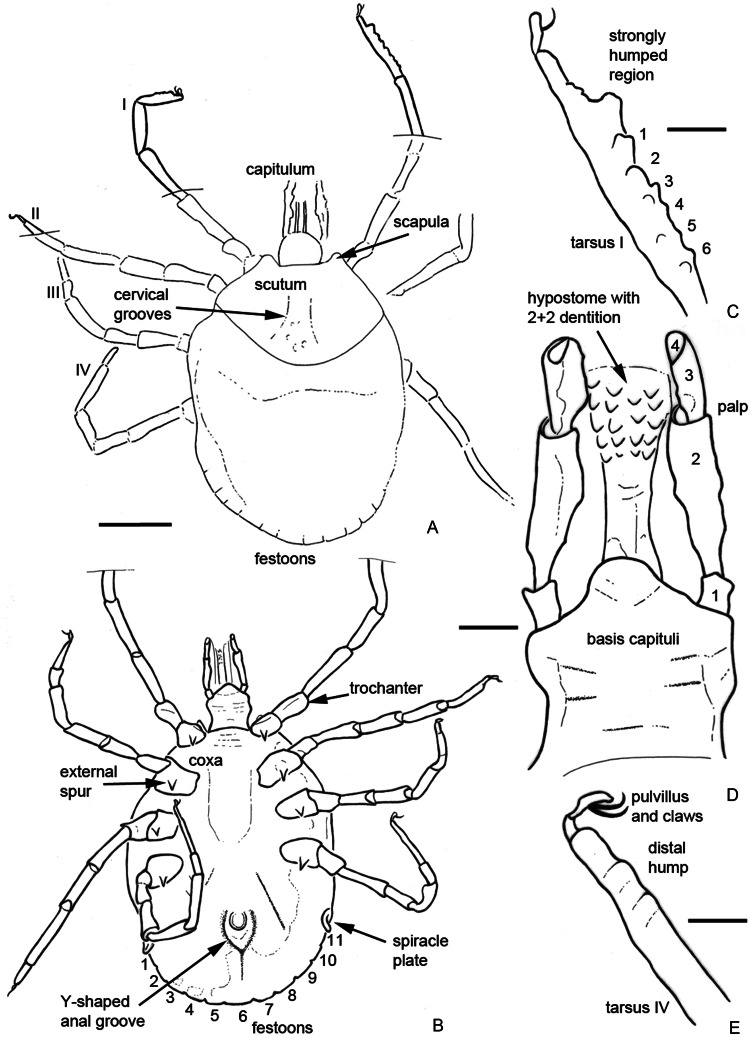
*Camera lucida* drawings of the specimen shown in Fig. 1. (A) Dorsal view. (B) Ventral view. (C) Details of leg I tarsus including humped distal region plus 6 additional lobes (numbered). (D) Details of capitulum; palpal articles numbered, note the elongate second article. (E) Details of leg IV tarsus with small hump. Legs numbered from I to IV. Scale bars equal 500 *μ*m (A, B) and 100 *μ*m (C–E).

*Idiosoma*: Ornamentation indistinct; body oval; length from apices of scapulae to posterior body margin 2013 *μ*m, breadth 1525 *μ*m; scutum width 1056 *μ*m (measured in middle of scutum), and 747 *μ*m (from scapula to edge); subtriangular with hints of a few central large punctuations; scapulae short and rounded (Fig. 2A), cervical grooves long, shallow and linear anteriorly and slightly diverging posteriorly, not reaching end of scutum; eyes equivocal, probably absent; 11 festoons ranging from 145 to 197 *μ*m in basal width (Fig. 1D); spiracle plates extruding noticeably from lateral body margins, 183 *μ*m long, 136 *μ*m wide (Figs 1D and 2B); anus visible, median, to the level of spiracle; anal groove Y-shape, close to anus with lateral arms reaching the upper limit of the anus and slightly converging (Fig. 2B), tail of the ‘Y’ does not reach middle festoon.

*Capitulum*: Length from apices to posterior margin of basis 725.2 *μ*m; basis capituli rectangular, 239.9 *μ*m long and 334.8 *μ*m broad, posterior margin straight, lateral margins straight, cornua absent, ventrally posterior margin straight, palpi elongated (Figs 1E and 2D), with length of 4 articles as follows: article 1, 79 *μ*m; article 2, 254.1 *μ*m – with external proximal side concave, while distal end of article 2 is noticeably wider, 1.86 times longer than third article – article 3, 136.5 *μ*m; article 4, 31.9 *μ*m; hypostome shorter than the palpi, length 182 *μ*m, width at base 97.7 *μ*m; hypostomal dentition 2/2 (Figs 1E and 2D), with 5 or 6 well-developed, rounded denticles in each file; porose areas absent.

*Legs*: Coxae I–IV with small external spur (Figs 1C and 2B); tarsus I with stout, strongly humped region distally (Figs 1F and 2C); 6 less prominent lobes present dorsally, decreasing in size in the direction of trochanter, no spurs, length 522 *μ*m; tarsus IV also ends in hump (Figs 1G and 2E); claws paired, slender, simple, slightly curved; with distinct pulvillus on all legs; Haller's organ small, located before the strongly humped region.

*Chaetotaxy*: Dorsally any setae (see below) were not visible, as the amber matrix bears many scratches hindering further examination.

**Fig. 3.**
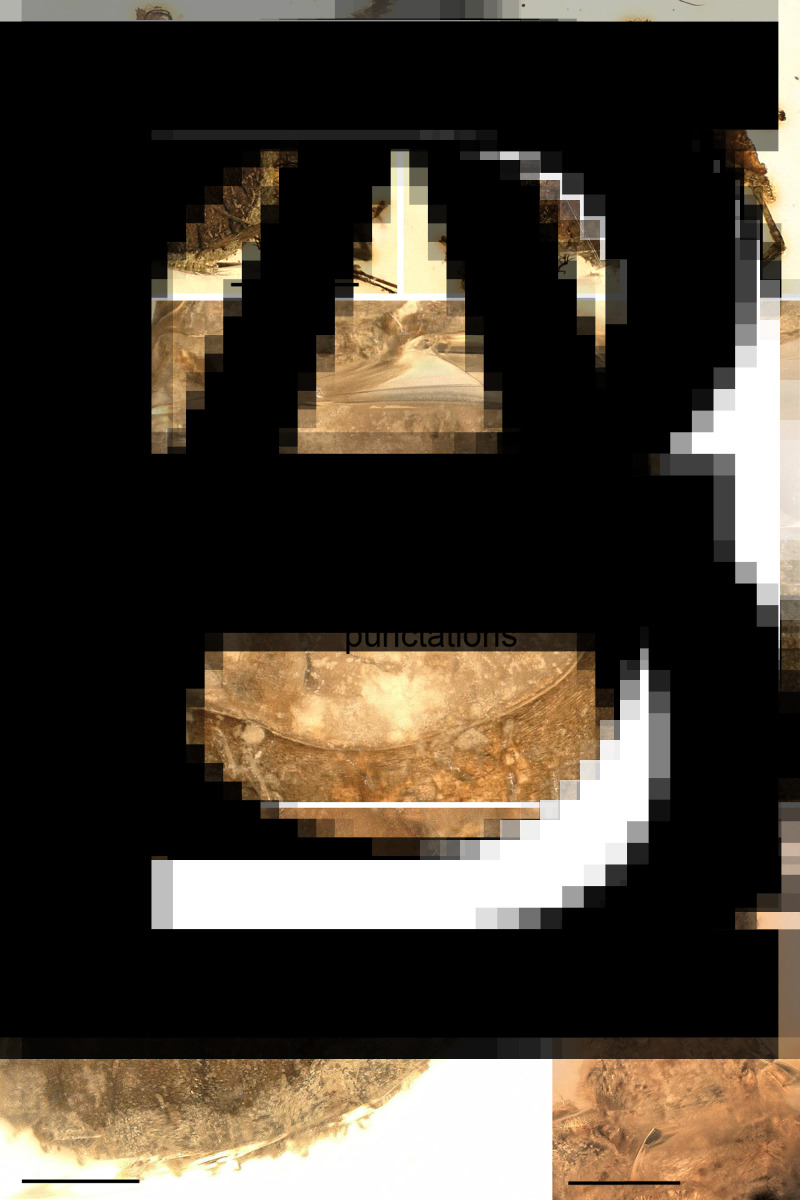
Paratype of *B. muelleri* sp. nov., P. Müller collection no. BUB1573, from Late Cretaceous (ca. 100 Ma) Burmese amber from Myanmar. (A) Dorsal overview. (B) Ventral overview. (C) Scutum showing large punctuations, cervical grooves (arrowed), but no evidence of eyes; note also several white, peg-like setae on the idiosoma. (D) Posterior idiosoma showing Y-shaped anal groove. (E) Capitulum. Scale bars equal 1 mm (A, B), 200 *μ*m (C–E).

*Description of the paratype*: BUB1573 is another unengorged nymph (Fig. 3); slightly smaller than the holotype.

**Fig. 4.**
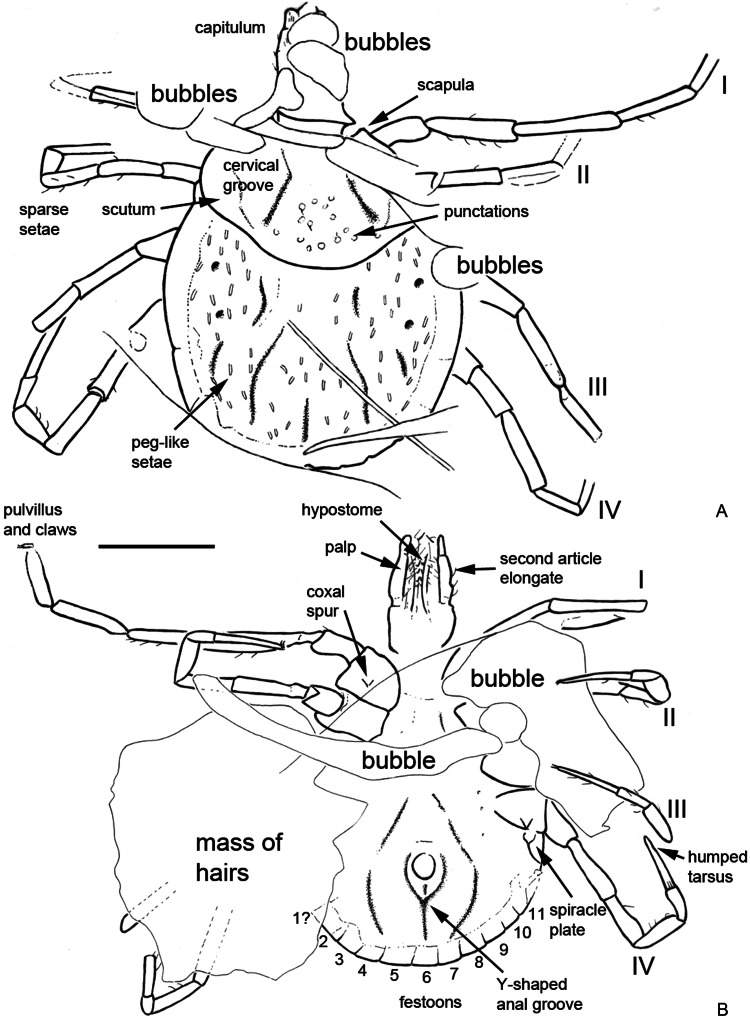
*Camera lucida* drawings of the paratype specimen shown in Fig. 3. (A) Dorsal view. (B) Ventral view. Legs numbered from I to IV. Scale bar equals 500 *μ*m.

*Idiosoma*: Body oval with integument leathery; length from apices of scapulae to the posterior body margin 1333 *μ*m, breadth 1207 *μ*m; scutum width 896 *μ*m (measured in middle of scutum), and 558 *μ*m (from scapula to edge); subtriangular with few central large punctuations (Fig. 3C); scapulae short and rounded, cervical grooves long, shallow and linear anteriorly and slightly diverging posteriorly, not reaching end of scutum (Figs 3C and 4A); eyes absent; 11 festoons (first indistinct); spiracle plates on lateral body margins (Fig. 4B), 134 *μ*m long, 88.8 *μ*m broad; anus visible, median, up to level of spiracle; anal groove Y-shaped (Figs 3D and 4B), close to the anus with lateral arms reaching upper limit of anus and slightly converging, tail of the ‘Y’ does not reach middle festoon.

*Capitulum*: Length from apices to posterior margin of basis 482.5 *μ*m; basis capituli rectangular, 143.8 *μ*m long and 282.6 *μ*m broad, posterior margin straight, lateral margins straight, cornua absent, ventrally posterior margin straight, palpi elongated, with length of 4 articles as follows: article 1, 43.5 *μ*m; article 2, 175.6 *μ*m – again with external proximal side concave and distal end of article 2 noticeably wider, 2.64 times longer than third article – article 3, 66.3 *μ*m, without external spur; article 4, 32.5 *μ*m; hypostome shorter than the palpi (Figs 3E and 4B), length 182 *μ*m, width at base 97.7 *μ*m; hypostomal dentition apparently 2/2 but not as clearly preserved as in holotype, at least 4 well-developed, rounded denticles in each file; porose areas absent.

*Legs*: Coxae with small external spur (not clearly visible on all legs); tarsus IV ends with slight hump (Fig. 4B); claws paired, slender, simple, slightly curved; with distinct pulvillus on all legs.

*Chaetotaxy*: Small hairs can again be observed on the legs (Fig. 4). Idiosoma covered dorsally by numerous, short, white, peg-like setae (Figs 3C and 4A) (68–83 *μ*m long and 19.8 *μ*m broad), but very few hairs observed on scutum; a few hairs visible ventrally.

*Remarks*: The 2 fossils described here as *Bothriocroton muelleri* sp. nov. are presumed conspecific, despite a slight difference in size, and represent a new hard tick genus for Burmese amber. They appear to lack eyes (Figs 1 and 2), and while blindness matches the condition in, e.g. *Compluriscutula vetulum* Poinar and Buckley, 2008 the pedipalps of *C. vetulum* differ from those in the new fossils. Specifically, in *C. vetulum* the 4th article of the pedipalp is long and ends with elongate terminal setae (cf. Fig. 2D here with its short article 4). Also the idiosoma in *C. vetulum* bears 13 festoons along its posterior margin (Poinar and Buckley, 2008, Fig. 3), while the new specimens have only 11 (Figs 1 and 2), all more or less the same size. The new taxon is thus the 4th Burmese amber tick species with 11 festoons; the others being *Cornupalpatum burmanicum* Poinar and Buckley, 2008, *Amblyomma birmitum* Chitimia-Dobler *et al*., 2017 and *Haemaphysalis* (*Alloceraea*) *cretacea* Chitimia-Dobler *et al*., 2018. Of these, *C. burmanicum* differs from the new fossil in having a unique extra claw on the penultimate (third) pedipalp article (Poinar and Brown, 2003, Figs 3 and 4), which is not seen in the new fossils (Fig. 2D). The absence of eyes in the new fossils is the strongest character for excluding *A. birmitum*. The other amber species in an extant genus, *H. cretacea*, has an elongate body, also with 11 festoons, and is eyeless, but unlike the new fossils it has the brevirostrate condition and has a dental formula with 8–10 denticles in a file.

### Comparisons with extant ticks

The *Amblyomma sensu* Horak *et al*. (2002) remains a controversial genus that includes ticks with quite different morphologies and lifestyles. Twenty eyeless *Amblyomma* species have been noted in the literature (Horak *et al*., 2002), of which 19 are now valid (Madder *et al*., 2010; Schachat *et al*., 2018), all of which potentially merit comparison with the new (blind) nymphs. The validity of these eyeless *Amblyomma* species as a group is still subject to debate and a wider reevaluation/reclassification of the entire *Amblyomma* genus may be necessary (Kaufman, 1972; Klompen *et al*., 1997, 2002). In detail, several *Amblyomma* species were originally placed in another genus, *Aponomma* Neumann, 1899, although the revision of Kaufman (1972) revealed this to be a heterogeneous group of eyeless ticks with a predilection for reptiles. Kaufman (1972) divided *Aponomma* into 3 species groups: (1) ‘typical’ *Aponomma*, including 15 species of reptile-associated ticks from the Afrotropical and Oriental zoogeographic regions, (2) ‘indigenous Australian’ species (now *Bothriocroton*; see below) and (3) 2 ‘primitive’ *Aponomma* species.

### *Robertsicus* and *Archaeocroton*

The ‘primitive’ *Aponomma* species *sensu* Kaufman (1972) has recently been elevated by Barker and Burger (2018) to new, monotypic genera, namely *Robertsicus* Barker and Burger, 2018 and *Archaeocroton* Barker and Burger, 2018. *Robertsicus* was erected for *Robertsicus elaphensis* (Price, 1958), formerly *Amblyomma elaphense* Price, 1958 that parasitizes the Trans-Pecos rat-snake in the Chihuahuan Desert of Mexico and south-eastern USA. Similar to the new fossils (Fig. 1E) this genus/species has a 2/2 hypostomal dentition, but differs in a scutum which is broader than long, but is cordiform, smooth and without cervical grooves (cf. Figs 2B and 4A of the new fossils), the setae and punctuations are minute and it has a very small, bluntly rounded spur on coxae I–IV (e.g. Keirans *et al*., 1985). In the new fossils the spurs are larger (Figs 1C and 2B). *Archaeocroton* was erected for *Archaeocroton sphenodonti* (Dumbleton, 1943). The 2 amber nymphs can also be excluded from *Archaeocroton* (see below) as the scutum here is sub-cordiform and the coxae have an external spur and a single subterminal spur on the trochanter (Kaufman, 1972). The new fossils lack spurs on the trochanter (Figs 2B and 4B).

### Eyeless *Amblyomma*

Identifying ‘*Amblyomma*-like’ nymphs is extremely difficult in most cases, even more so with fossils. So far, there are no workable keys, and there are no established diagnostic characters for nymphs of either *Amblyomma* or *Bothriocroton*. However, a comparison with known eyeless *Amblyomma* nymphs (as the new fossil is eyeless) and with described *Bothriocroton* nymphs are given in the sections below.

Several characters exclude the eyeless *Amblyomma* species which corresponded to the ‘typical’ *Aponomma* species *sensu* Kaufman (1972). Nymphs of this species group are characterized by an anal groove being present and embracing the anus posteriorly; eyes absent; festoons present and numbering 11; basis capituli variable in form but often subrectangular or subtriangular; palpi elongate and constricted proximally, with article 2 being especially long and with reptiles as hosts (Horak *et al*., 2002; Klompen *et al*., 2002; Barker and Murrell, 2004; Beati *et al*., 2008).

Excluding the 2 ‘primitive’ ticks (see above) which are now in their own genera, the eyeless *Aponomma sensu* Kaufman (1972) is distributed as follows. There are 7 Afrotropical species: *Amblyomma arcanum* Karsch, 1879, *Amblyomma exornatum* Koch, 1844, *Amblyomma flavomaculatum* Lucas, 1846, *Amblyomma inopinatum* Santos Dias, 1989, *Amblyomma latum* Koch, 1844, *Amblyomma orlovi* Kolonin, 1992 and *Amblyomma transversale* Lucas, 1845. *Amblyomma transversale* has recently been reinstated as the separate genus *Africaniella* Travassos Dias, 1974 (Hornok *et al*., 2020). There is also 1 Afrotropical/Oriental species (*Amblyomma gervaisi* Lucas, 1847), 3 Oriental species (*Amblyomma crassipes* Neumann, 1901, *Amblyomma fuscolineatum* Lucas, 1847 and *Amblyomma pattoni* Neumann, 1910), 3 Oriental/Australasian species (*Amblyomma fimbriatum* Koch, 1844, *Amblyomma trimaculatum* Lucas, 1878 and *Amblyomma varanense* Supino, 1897) and 3 purely Australasian species (*Amblyomma komodoense* Oudemans, 1928, *Amblyomma kraneveldi* Anastos, 1956 and *Amblyomma soembawense* Anastos, 1956). Any of these could potentially be related to the new amber fossils – which could be taken as evidence for their inclusion in the ‘eyeless’ *Amblyomma* assemblage – but as detailed below these modern species are either known only from adults or differ from the new material.

### Eyeless *Amblyomma* in the Afrotropics

*Amblyomma arcanum* and *A. inopinatum* were accepted as valid species by Guglielmone and Nava (2014) and only adults are known (Guglielmone *et al*., 2014), which makes it difficult to compare directly to the new amber species at the nymphal stage. For *A. orlovi* it is not clear whether this is a valid species and is known only from the female (Guglielmone *et al*., 2014). *Amblyomma exornatum* is known from various stages and differs from the new fossils in having a hypostome dentition of 3/3 (2/2 in the fossils: Figs 1E and 2D), teeth continuing posteriorly as crenulations and a small corona distally, and tarsus I with a small distal dorsal hump (Theiler, 1945*a*); as opposed to the large hump seen in the fossils (Figs 1F and 2C). Based on personal observations of *A. flavomaculatum* nymphs held in the Museum für Naturkunde Berlin, observations include: a hypostome dentition of 3/3 with teeth in 6–7 lines, a rosette visible distally, the cervical grooves are short, deep and arched, the setae and interstitial punctuations are small and regularly distributed and the nymphal tarsus is similar to that of the female with less prominent protrusions (see also Saratsiotis, 1972).

*Amblyomma latum* is a senior synonym of several species and is characterized by a spot of red pigmentation in the eye region, cuticle which is thin and transparent with no hairs present. Again, comparative material (labelled as a synonym: *Amblyomma leave* Neumann, 1899) in the Museum für Naturkunde collection was examined. The hypostome has a 2/2 dentition (like the fossils) but with a corona, coxa I has an external spur sharper and longer than the internal one (only 1 spur in the fossils: Figs 1C and 2B), coxae II–IV have 1 median spur; tarsus I long; tarsi II–IV taper fairly gradually from a slight median swelling in some instances this swelling is large enough to produce the effect of a hump (Theiler, 1945*b*), but not to the same extent as in the fossils (Figs 1F and 2C). Finally, *A. transversale* has abdominal skin showing fine transverse striations, but hairs appear to be absent. The coxae have a single spur, tarsus I show well-pronounced false articulation, the distal portion with a large hump and 1 or 2 ventral spurs (Theiler, 1945*b*).

### Eyeless *Amblyomma* from other regions

Taxa occurring outside (wholly or in part) of the Afrotropical region are potentially closer in terms of biogeography to possible Australasian-derived fossils. *Amblyomma gervaisi* ranges across the Afrotropical to the Oriental region. The species is glabrous, with a scutum longer than wide, subtriangular and non-ornamented (punctate in the fossils: Fig. 4A). Its cervical grooves are quite long and the tarsus is without a spur (Neumann, 1899). The purely Oriental species, *A. crassipes* has a subcordiform scutum and, similar to the fossils, has large punctuations over the entire surface. Unlike the fossils (Figs 1E and 2D), the hypostome has a 3/3 dentition at its base and a corona. Coxa I has 2 spurs and coxae II–IV 1 spur. Tarsus I shows a distinct swelling (Theiler, 1945*a*), but not the strong hump as in the fossils (Figs 1F and 2C). *Amblyomma crassipes* is similar to *A. fuscolineatum* (see below), but is provisionally considered valid because scant material is available for examination and the types have not been compared (Guglielmone *et al*., 2010).

*Amblyomma fuscolineatum* Lucas, 1847 is variously recorded in the literature (see Guglielmone and Nava, 2014). The description of the nymph in Schulze (1936) is confusing, but the hypostome has a 3/3 dentition and the cervical groove is divergent and reaches the end of the scutum. *Amblyomma pattoni* is a senior synonym of *Aponomma pseudolaeve* Schulze, 1935; cf. Kaufman (1972) and Camicas *et al*. (1998). The description in Schulze (1935) is very brief; noting that the nymph looks like the female except that the hypostome is different. Thus the hypostome dentition is 2/2 and punctuations are generally absent with just a few punctuations on the scapula (Schulze, 1935).

Other species occur from the Oriental through to the Australasian region. *Amblyomma fimbriatum* is a senior synonym of several taxa (see Guglielmone and Nava, 2014). It has the scutum postero-lateral margins straight or slightly convex, several, large, scattered punctuations and a few small ones, cervical grooves ending in short, ill-defined depressions, a hypostome dentition of 3/3 (2/2 in the fossils), coxae each with a single short spur (similar to the fossils) and tarsi humped but without spurs (Roberts, 1953). *Amblyomma trimaculatum* has a scutum with the postero-lateral margins slightly convex, punctuations relatively large and distributed mainly laterally, the hypostome dentition is again 3/3 and the tarsi are humped, but without spurs (Roberts, 1953). Finally, from the Australasian region, *A. komodoense* and *A. kraneveldi* only adults are known (Guglielmone *et al*., 2014) precluding direct comparison to the new fossil nymphs. *Amblyomma soembawense* Anastos, 1956 is only known from adults and the larva: the nymph is unknown (Guglielmone and Nava, 2014).

### Assignment to *Bothriocroton*

In the absence of a strong match between the 2 amber nymphs and any of the ‘eyeless’ *Amblyomma* species (see above), the next taxon to consider is *Bothriocroton*. This subgenus was raised by Keirans *et al*. (1994) for *Aponomma glebopalma* Keirans, King and Sharrad, 1994 and subsequently expanded (Klompen *et al*., 2002) to include the rest of Kaufman's (1972) ‘indigenous Australian’ *Aponomma* ticks, with *Bothriocroton* also placed in its own subfamily: Bothriocrotoninae. The ‘indigenous Australian’ *Aponomma* species were recognized by Kaufman (1972) on the following character combinations: a single subterminal spur on the trochanter (absent in all other *Aponomma* and in *Bothriocroton glebopalma*, and also absent in the new fossils) and lateral grooves on the scutum of the male partial or complete – absent in ‘typical *Aponomma*’, *R. elaphensis*, and *B. glebopalma* – but present in *A. sphenodonti*. Thus absence of eyes, elongate palps, a subpentagonal basis capituli shape, coxae with 2 spurs in all instars and trochanters with a single subterminal ventral spur – albeit absent in *B. glebopalma* (see e.g. Klompen *et al*., 2002) – are morphological features that can be considered characteristic for the living *Bothriocroton* species. Spiracle plates extruding from the lateral border of the idiosoma anterior to the first festoon in *Bothriocroton oudemansi* (Neumann, 1910) were first described by Neumann (1910) and are present in the new fossil. The large wax glands laterally near setae s6, Md3 of Clifford *et al*. (1961), and anterior to the first festoons (Klompen *et al*., 1996) in the larvae are also promising diagnostic characters, but cannot be tested in the present fossil material.

**Fig. 5.**
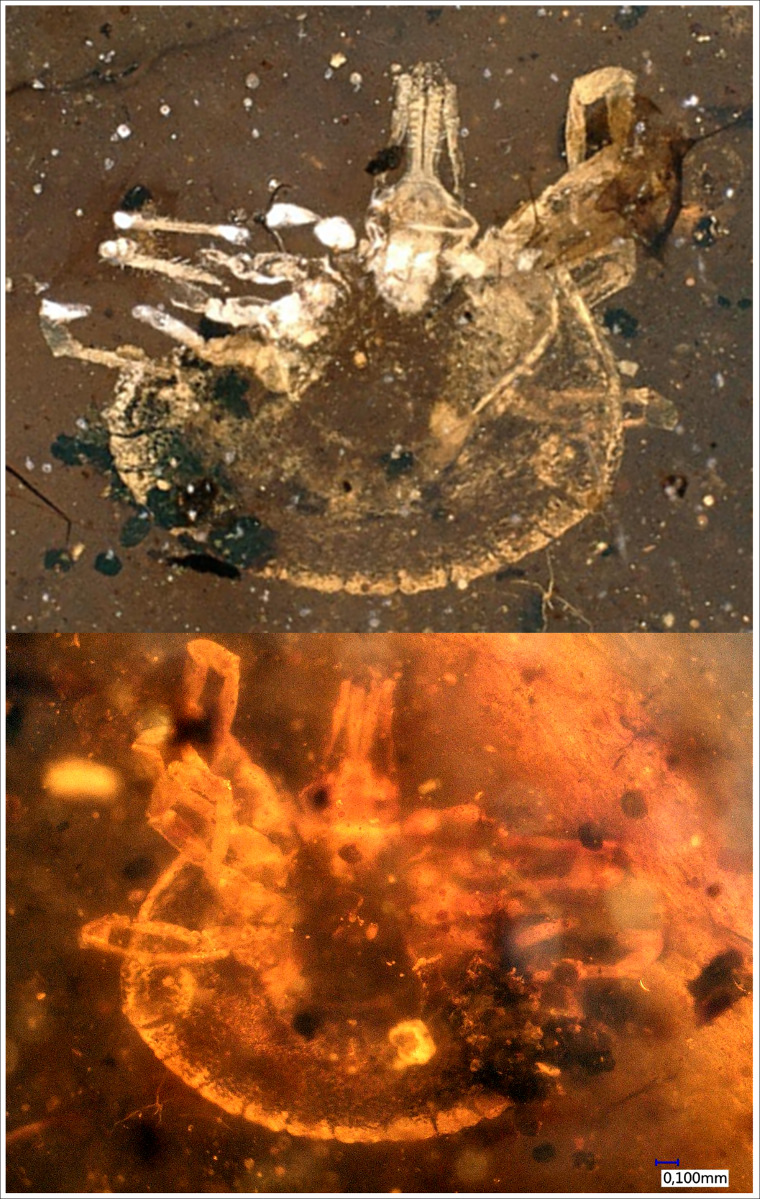
Holotype and only known specimen of the hard tick *Archaeocroton kaufmani* sp. nov. (Ixodida: Ixodidae) in the Museum für Naturkunde, Berlin (accession number MB.A. 4452).

The new Burmese amber nymphs are tentatively assigned to *Bothriocroton* based on a combination of: (1) the absence of eyes, (2) the second article of the palps being longer than the third (Figs 1E and 2D), (3) a dental formula of 2/2 in a file (Figs 1E and 2D), (4) coxae I–IV with obvious external spurs (Figs 1C and 2B), (5) the absence of a trochanter spur, (6) tarsus I with a stout, strong hump and 6 small dorsal protrusions (Figs 1F and 2C) and tarsus IV also ending with a hump (Figs 1G and 2E) and (7) spiracle plates extruding from the lateral border of the anterior to the first festoon (Fig. 1B and D). The 7th character could place the new fossil tick close to some Asian *Amblyomma*, species (see above) which, at least in the adult stage, have 3 lobes close to the hump: for example, *Amblyomma supinoi* in Voltzit and Keirans (2002). It is also similar to some *Ornithodoros* species – albeit in a different family, Argasidae – which also present prominent dorsal lobes, including bipartite lobes, which are used in species diagnoses (see e.g. Bakkes *et al*., 2018).

*Bothriocroton* is, numerically, a small tick genus today with only 7 extant species and no division into subgenera (Barker *et al*., 2014; Guglielmone *et al*., 2014). In addition to the type species (*B. glebopalma*), 4 of its species were traditionally referred to *Aponomma*: *Bothriocroton auruginans* (Schulze, 1936), *Bothriocroton concolor* (Neumann, 1899), *Bothriocroton hydrosauri* (Neumann, 1899) and *Bothriocroton tachyglossi* (Roberts, 1953). Note that *B. tachyglossi* was considered a synonym of *B. hydrosauri*, but there is convincing evidence for its validity (Guglielmone *et al*., 2010). The 2 remaining species are *B. oudemansi* (Neumann, 1910) – at one time considered a synonym of *B. concolor*, although there is sound evidence for its validity (Guglielmone *et al*., 2010) – and *Bothriocroton undatum* (Fabricius, 1775) (=*Aponomma decorosum* Koch, 1867).

Morphology shows *Bothriocroton* closely related to *Amblyomma* and the general leg and palpal chaetotaxy resembles that of *Amblyomma sensu lato*. *Bothriocroton* species are generally rather large ticks with long mouthparts: the longirostra condition. Iridescent ornamentation on the scutum is absent, but the living species *B. glebopalma* and *B. undatum* have white ornamentation, which could not be observed in the new fossils. *Bothriocroton* ticks can also be recognized by the adult hypostomal dentition, which is either in a 2/2 or 3/3 arrangement; with the internal row much smaller than other rows. The scutum is also broader than long. In females and nymphs, conspicuous posterolateral indentations formed by the confluence of larger punctuations are present.

### Comparisons to living *Bothriocroton* species

The new amber fossils appear to be morphologically closest to the extant species *B. undatum*, especially with respect to the dental formula, the coxal spurs and stout, strong hump which is very evident on tarsus I and dorsal lobes which are not so prominent (Figs 1F and 2C), plus a small hump on tarsus IV (Figs 1G and 2E). The fossils also share with *B. oudemansi* the same dental formula and coxal spurs (see e.g. Beati *et al*., 2008); however, tarsal humps are lacking in this modern species. The scutum in the new fossils is subtriangular, broader than long, with its postero-lateral margins slightly convex. It is larger than the scutum in both *B. undatum* and *B. oudemansi*. The cervical grooves form long, deep depressions (Figs 2A and 4A), which broaden and become shallow and divergent, but do not reach the posterior scutal margin. It may also be noted that *B. glebopalma* has a deeply pitted and pilose scutum in both the adult and immature stages, unlike the other eyeless *Amblyomma* species previously placed in *Aponomma* (Keirans *et al*., 1994).

### Systematic palaeontology for the *Archaecroton*

*Emended diagnosis*: Metastriate ticks with body sub-circular; 11 festoons; basis capitulum lateral angles produced into sub-conical processes; hypostome dentition 2/2 without corona; spiracle plates sub-circular; tarsi I–IV with pseudo-articulation at 1/3 of its length.

Family Ixodidae Murray, 1877

Genus *Archaeocroton* Barker and Burger, 2018

*Archaeocroton kaufmani* Chitimia-Dobler, Mans and Dunlop sp. nov.

*Etymology*: After T. S. Kaufman who first recognized the distinctness of the modern species now placed in *Archaeocroton*.

*Material*: Holotype from Burmese amber, Myanmar. Late Cretaceous (Cenomanian) in the Museum für Naturkunde, Berlin (accession number MB.A. 4452).

*Diagnosis*: Fossil *Archaeocroton* in which cornuae are extremely broad; spiracle plates comma shape; hypostome dentition 2/2 without corona; 11 festoons; tarsi I–IV with pseudo-articulation to the 1/3 of its length (Supplementary Fig. 1); tarsus IV ends with a dorso-apical hook.

*Description*: Female.

**Fig. 6.**
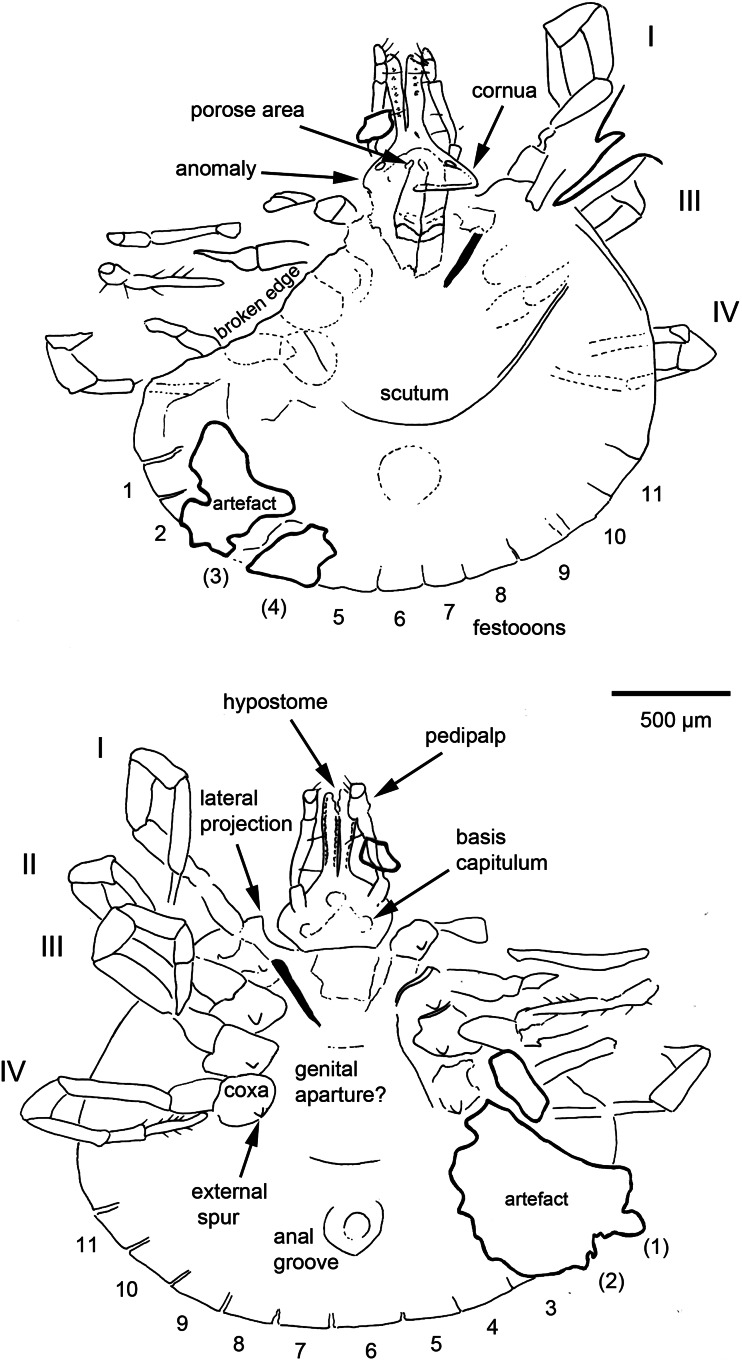
Interpretative *camera lucida* drawings of the specimen shown in Fig. 5. Legs numbered from I to IV and festoons numbered from 1 to 11.

**Fig. 7.**
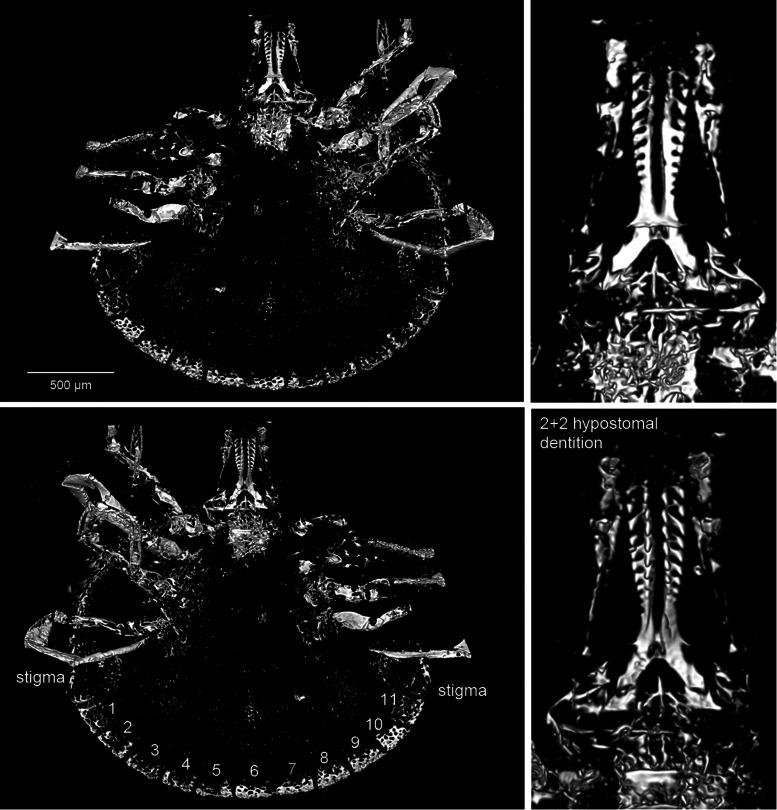
CT images of the holotype (penetrating the artefacts) and emphasizing again the presence of 11 festoons (left both above and down). Additionally highlighting the outline of the stigma (left down), chelicera structure (right above) and the 2 + 2 dentition of the hypostome (right down). The volume renderings especially emphasize air-filled cavities in the amber fossil.

*Idiosoma*: Body subcircular, slightly elongate, widest posteriorly, integument leathery; length from middle of scutum to posterior body margin 1390 *μ*m. Maximum width (measured in middle, behind third legs) 1984 *μ*m; scutum 1156 *μ*m wide (measured in middle), or 837 *μ*m long (from middle to edge); sub-cordiform, with few spare punctuations (Figs 5 and 6); scapulae short and rounded. Cervical grooves not visible (Figs 5 and 6). Posterior margin of idiosoma with 11 visible festoons (width 176–229 *μ*m) (Figs 5 and 6), no grooves separate festoons from the rest of idiosoma. Anus visible, median; anal groove semi-circular, contouring behind anus (Figs 5 and 6). Genital aperture not clearly visible, apparently located approximately between coxae II and III. Stigmas best seen in CT scans (Fig. 7), comma-shaped with round macula; left stigma 392 *μ*m long and 265 *μ*m wide, right stigma 403 *μ*m long and 275 *μ*m wide.

*Capitulum*: Length from apices to posterior margin of basis 569 *μ*m. Basis capitulum triangular dorsally with lateral angles produced into sub-conical processes, length 452 *μ*m, maximum width 188 *μ*m, more than twice as broad as long; posterior margin straight, cornuae extremely broad and very blunt. Ventrally sub-triangular, lateral projection distinct and appears acute in ventral view; auriculas indistinct; porose areas only slightly visible, but sub-circular, located centrally on basis capituli. Palpi elongate; 4 articles with lengths: article 1, 82 *μ*m; article 2, 280 *μ*m (with external proximal side concave); article 3, 133 *μ*m; article 4 could not be measured. Hypostome somewhat shorter than adjacent palpi (Figs 5 and 6); hypostomal dentition 2/2. The external left file has 10 well-developed denticles, the external right file has only 9; internal files have 7 smaller denticles; apical corona absent. Chelicerae well-developed, equal in length to hypostome, with minute dorsal denticles on internal side of the unmovable fingers and with sawdust appearance due to the 6 kinds of teeth (Fig. 7).

*Legs*: Coxae I–IV without internal spurs; external spur on coxae I–IV is small. Tarsus I taper distally and has a pseudo-articulation at 1/3 of its length; tarsi IV 520 *μ*m (length of tarsus 190 *μ*m, length of tarsus 330 *μ*m) with pseudo-articulation at 1/3 of its length and ending with a dorso-apical hook; tarsi II and III with pseudo-articulation at 1/3 of its length.

*Chaetotaxy*: Short hairs observed on all leg articles. Palps bear long seta on internal (mesal) side of second article, and 3 long internal setae on third article, plus 1–2 setae on external side of the second and third articles (Figs 5 and 6).

*Anomaly*: Basis capitulum reveals an abnormality on the left side (Figs 5 and 6). Deformation occurs before sub-conical process and goes a little below the basis capitulum. Ventrally lateral projection not completely developed, appearing more round than angular. Anomaly also reaches basis of hypostome, where right external file has only 9 denticles (not 10 as per left part) and is thicker than normal.

*Remarks*: The tuatara tick was originally described as *Aponomma sphenodonti* Dumbleton, 1943 and was regarded as a ‘primitive *Aponomma* species’ *sensu* Kaufman (1972), who hinted that it may warrant a separate genus. *Aponomma* Neumann, 1899 was subsequently synonymized with *Amblyomma* by Klompen *et al*. (2002) (see also above), while Barker and Burger (2018) confirmed Kaufman's earlier intuition and recognized the tuatara tick as the sole representative of a new genus, *Archaeocroton*, so named for retaining several apparently plesiomorphic features. The body of the new female amber specimen is sub-circular, slightly wider than long, widest posteriorly and has a leathery integument. In this sense it is similar to the previously described Burmese amber fossil *A. birmitum* as well as some living members of *Amblyomma* (Voltzit and Keirans, 2003), to *B. oudemansi* (Beati *et al*., 2008), *R. elaphensis* and to *A. sphenodonti* (see e.g. Barker and Burger, 2018). The new fossil lacks eyes, and in this sense it is similar to the Burmese amber species *C. burmanicum* (Poinar and Brown, 2003), *C. vetulum* (Poinar and Buckley, 2008) and *H. cretacea* (Chitimia-Dobler *et al*., 2018).

The number of festoons around the posterior margin of the idiosoma can be a useful diagnostic character in metastriate ticks, both at the genus and species levels. *Hyalomma* have 5, *Haemaphysalis* have 9 or 11 and *Amblyomma* have 11 (Neumann, 1911; Nuttall *et al*., 1911; Lindquist *et al*., 2016). The central festoon is usually positioned directly behind the anus (Clifford and Anastos, 1960). The new fossil has 11 well-defined festoons (Figs 5–7) and no groove separates the festoons from the rest of the idiosoma. The number of festoons and the shape of the stigmata are thus morphological features similar to the Burmese amber fossil *A. birmitum*. The new fossil has the genital aperture located between coxae II and III. This would be similar to fossil *A. birmitum* (Chitimia-Dobler *et al*., 2017) and to some modern *Amblyomma* species coming from former Gondwanan regions, and also to *B. oudemansi* (Kaufman, 1972; Beati *et al*., 2008) and to *A. sphenodonti* (Barker and Burger, 2018). By contrast, other species have the genital aperture located between coxae II (Voltzit and Keirans, 2003; Voltzit, 2007), except for 3 Neotropical *Amblyomma* species which have the genital aperture between coxae III: namely *Amblyomma cruciferum* Neumann, 1901, *Amblyomma darwini* Hirst and Hirst, 1910 and *Amblyomma humerale* Koch, 1844 (Voltzit, 2007).

The key character here is the shape of the basis capitulum, which is very distinctive in the new fossil. It is triangular dorsally with lateral angles produced into sub-conical processes and extremely broad with very blunt cornuae, while ventrally it is sub-triangular; the lateral projections are distinct and appear acute with indistinct auriculas. A basis capitulum of this form is very rare among the extant ixodid females. This triangular shape dorsally and sub-triangular with lateral projections ventrally is specific for females of *A. sphenodonti*, and for nymphs and larvae of *Hyalomma*, but not for the adults (Apanaskevich and Horak, 2008). Some extant *Amblyomma* species can have a (sub)triangular basis capitulum, as do the extinct Burmese amber genera *Cornupalpatum* and *Compluriscutula* (Poinar and Brown, 2003; Poinar and Buckley, 2008), however, in all these cases the basis capitulum is not as broad as in the current fossil.

**Fig. 8.**
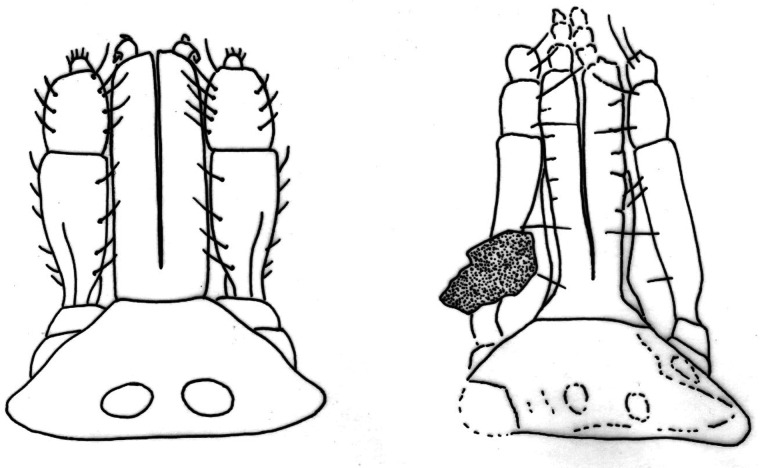
Comparative sketches of the distinctively triangular basis capitulum in dorsal view in the extant tuatara tick, *Archaeocroton sphenodonti* Dumbleton, 1943 (left), and the new amber species *A. kaufmani* sp. nov. (right), with the special chelicera structure. Not to scale.

The denticles on the hypostome are usually arranged in parallel longitudinal rows, or files, and the dentition formula indicates the number of files on each side of the midline of the hypostome. For example, 2/2 indicates the presence of 2 files on each side. The lateral-most row is designated as file 1. The relative size of the denticles differs characteristically between different species of ticks, such that the number of teeth in each file is a useful diagnostic character. The lateral file generally has denticles at least as large as any present elsewhere on the hypostome (Lindquist *et al*., 2016). In the new fossil, the hypostome tooth columns have a 2/2 arrangement, distributed along the whole length of the hypostome (Figs 5 and 6), with 10 well-developed teeth in the external file and 7 smaller teeth in the second, without corona. All modern Asian, African and Neotropical *Amblyomma* adults have a 3/3 or 4/4 tooth arrangement, or even 5/5 in females of *Amblyomma clypeolatum* Neumann, 1899 (Voltzit and Keirans, 2002, 2003; Voltzit, 2007). In a few modern species the internal files are also less developed than the external ones: *A. sphenodonti* (Barker and Burger, 2018), *B. tachyglossi* (Roberts, 1953) and *A. transversale* (Theiler, 1945*b*). In *A. sphenodonti* the hypostome tooth columns have a 2/2 arrangement, with 5 or 6 teeth in a file and corona (Dumbleton, 1943; Kaufman, 1972). The chelicerae structure is unique for ixodid ticks, none of the extant tick species is known to have a sawdust appearance on the internal side of the unmovable fingers (Fig. 8).

In the amber fossil the stigma is comma-shaped, with round macula and about 392–403 *μ*m long. In *A. sphenodonti* the stigma is sub-circular and 230 *μ*m long, slightly angulate at a posterior dorsal angle (Dumbleton, 1943). Tarsus IV (527 *μ*m) of the new fossil has a pseudo-articulation at 1/3 of its length and ends in a dorso-apical hook. Tarsi II and III have also a pseudo-articulation at 1/3 of its length, but no dorso-apical hook. Pseudo-articulation of tarsi I and IV is also characteristic for *A. sphenodonti*. The dorso-apical hook in the new fossil is not present on other ixodid species and represents a useful diagnostic character; see above. Usually, *Amblyomma* adults have 1 or 2 ventro-apical hook(s) (Kaufman, 1972; Voltzit and Keirans, 2003) as do some *Dermacentor* species (Filippova, 1997). Taken together, the observed morphological characteristics suggest that the new fossil is closely related to the tuatara tick *A. sphenodonti* and can also be placed in *Archaeocroton*.

### Anomaly in the basis capitulum

The anomaly observed in the basis capitulum with a discrepancy in the number of teeth between the left and right files is the first example recorded for a tick in amber. It can be classified as an exoskeleton anomaly. A number of biological or non-biological factors may cause morphological abnormalities in ticks: (1) somatic or germinal mutations, (2) injury, (3) exposure to chemical agents, (4) environmental stress, (5) host resistance to tick infestation or (6) blood-feeding on unusual hosts (Feldman-Muhsam, 1948; Campana-Rouget, 1959*a*, 1959*b*; Latif *et al*., 1988; Guglielmone *et al*., 1999; Dergousoff and Chilton, 2007; Nowak-Chmura, 2012). Zharkov *et al*. (2000) considered that the exoskeleton abnormalities in *Ixodes* tick are greatly dependent on anthropogenic pressure, and it may be the most reliable way of biomonitoring the environment pollution level.

### Scutum pattern and dental formula

No *Amblyomma* species have been described thus far with white spots related to colour patterns on the festoons, even more on all festoons and the side of the scutum. Among hard ticks, many species of Metastriata have intricate ornamentation on the scutum and/or festoons that is often used as a taxonomic character. However, the biological function(s) of this ornamentation remains unclear (Schachat *et al*., 2018). The scutum pattern of the fossil is similar to *A. sphenodonti* (Barker and Burger, 2018). In fact, the white pattern on the scutum and festoons is related to the parts of extant females where the purple zones are not observed. In addition, the new fossil shares with *A. sphenodonti* the shape of the basis capitulum and the shape and position of the porose areas (cf. Barker and Burger, 2018). *Amblyomma birmitum*, the first fossil female, has also a 2/2 dentition but the teeth are of the same size (Chitimia-Dobler *et al*., 2017). Some extant species have the hypostome dentition of the fossil female, e.g. *R. elaphensis* has a 2/2 hypostomal dentition (although the hypostome figured has a single supernumerary tooth between files 1 and 2 on the left side of the hypostome as viewed from above) and the hypostome ends with a denticle corona (Keirans *et al*., 1985), while *B. oudemansi* also has the same dental formula (Beati *et al*., 2008).

## Discussion

All living *Bothriocroton* ticks are restricted to the Australian faunal region while the single living *Archaeocroton* species is only found in New Zealand. The new Burmese amber fossils are thus not only the oldest putative records of these 2 genera, but also the only records outside their current biogeographical areas. An obvious question is whether the modern representatives are relicts of previously much wider distributions. Alternatively, are these Australasian genera Gondwanan in origin, with lineages that migrated into the area that became the Burmese amber forest prior to the mid-Cretaceous? Burmese amber was deposited on the so-called West Burma terrane which is presumed to have rifted from northern Australia at some point in Earth history. It forms part of the Incertus Arc which in some models is thought to have formed in the Late Jurassic (about 155 Ma) and was potentially linked to northern Australia and India *via* the Woyla Arc (see e.g. Hall, 2012). This connection may have provided a short window of land bridges for colonization of the Burma terrane by Gondwanan faunal elements, before the bridges were lost at the beginning of the Cretaceous (ca. 140 Ma). By the time of amber deposition (ca. 100 Ma) the Burmese amber forest was probably on an island (e.g. Westerweel *et al*., 2019), which eventually collided with southeast Asia.

This scenario would be consistent with *Bothriocroton* and *Archaeocroton* originating in Gondwana, with some lineages migrating onto the West Burma terrane and surviving until at least the mid-Cretaceous. Similarly, it explains the presence of an Australasian-like *Ixodes* in Burmese amber reported by Chitimia-Dobler *et al*. (2022). Mans *et al*.'s (2019) estimate of (maximally) 186 Ma for the radiation of metastriate ticks and 164 Ma for the divergence of the main metastriate lineages is well before the date at which the land bridges may have been lost (140 Ma). In other words, metastriates in Gondwanan territories may have diversified into the current genera just prior to the time when they could have still made their way onto the West Burma terrain. If this was not the case, and if the Burmese amber fauna were to be shown to be predominantly Laurasian in origin, the disjunct distribution for *Bothriocroton* and *Archaeocroton* in Burmese amber and in Australia and New Zealand, respectively needs explanation. Either a very broad former distribution across Asia and Australasia needs to be explained, or a more recent, probably post-Cretaceous, migration of these ticks from Asia to Australasia, plus the extinction of the genera in Asia needs to be inferred.

An interesting parallel case of Burmese amber hosting an endemic New Zealand taxon is the beetle genus *Cyclaxyra* Broun, 1893 (Cyclaxyridae) (Wu *et al*., 2018). Further examples of Burmese amber beetles which are morphologically very similar to living relatives restricted to an austral distribution (e.g. Australia, New Zealand, southern South America) were discussed by Cai *et al*. (2019 and references therein). They suggested that the modern populations may be relicts of a previously much wider distribution.

### Hosts of the living relatives

Examining the hosts of modern *Bothriocroton* and *Archaeocroton* species may also be informative for reconstructing the evolutionary history of these genera. *Bothriocroton hydrosauri* and *B. tachyglossi* have been mostly collected from the short-beaked echidna, or spiny anteater, *Tachyglossus aculeatus* (Shaw, 1792) (Monotrema: Tachyglossidae). In detail, Andrews *et al*. (2006) reported the 2 species from Wowan, Queensland, Boompa, Monto, Gladstone, Woolooga, Biloela, Rockhampton, Emu Park, Belmont, Yeppoon, St Lawrence, Mackay, Marian; from cattle in Dululu, Mackay, Degilbo and from an unknown host in Woolooga, Queensland. Putative specimens of ‘*A. hydrosauri*’ from a snake from the Darling Downs proved, on re-examination, to be *B. undatum*. In summary, no specimens of *B. hydrosauri* or *B. tachyglossi* were seen from reptiles in Queensland or from echidnas in southern Queensland (Andrews *et al*., 2006). *Bothriocroton glebopalma* has been described from the black-palmed rock monitor lizard *Varanus glebopalma* Mitchell, 1955 and the Kimberly rock monitor *Varanus glauerti* Mertens, 1957 in Western Australia and the Northern Territory. Some specimens were collected from formalin-preserved hosts, revealing that all stages of *B. glebopalma* parasitize monitor lizards (Keirans *et al*., 1994). *Bothriocroton glebopalma* was found in an area where *A. fimbriatum*, a parasite of monitor lizards and various species of snakes, was the only known member of the genus found in both regions mentioned above (Keirans *et al*., 1994). However, the same study also described *Amblyomma glauerti* Keirans, King and Sharrad, 1994 that occur in the same regions and parasitize the same hosts.

The living species which best matches the new fossils, *B. undatum*, has also been recorded from echidnas, snakes and monitor lizards (e.g. Roberts, 1964). In a wider context, Chitimia-Dobler *et al*. (2018) reviewed parameters in the fossil record for host–parasite co-evolution in ticks. It is conceivable that *B. muelleri* fed on dinosaurs or reptiles in the Burmese amber forests similar to related *Bothriocroton* lineages in Australasia. The Cretaceous–Palaeogene extinction event may have caused extant *Bothriocroton* species to undergo host switches to monotremes or to remain with reptiles.

A fossil assignable to *Archaeocroton* is of particular interest in that the only living species is hosted by the tuatara. The host lives in borrows associated with the fairy prion bird, *Pachyptila turtur* (Kuhl, 1820). Although this bird has been found infested with *Ixodes auritulus* Neumann, 1904, it has never been found carrying *A. sphenodonti*, which suggests that the tuatara tick is quite host-specific (Dumbleton, 1943). The tuatara is, of course, the only living representative of the Rhynchocephalia. This clade is the sister group of Squamata (i.e. lizards and snakes), many of which host species of *Amblyomma*. As such, a tick with several plesiomorphic characters is associated with a reptile popularly considered to be a ‘living fossil’ (Cree, 2014). In fact, the living fossil moniker has been challenged, or at least requires careful definition, as the tuatara belongs to a previously much more diverse lineage (Rauhut *et al*., 2012; Herrera-Flores *et al*., 2017) with rhyncocephalians originating in the Triassic and diversifying in the Jurassic. The new amber fossil may have also used a rhyncocephalian host. Although diversity dropped after the Jurassic, several rhyncocephalian lineages were still present during the mid-Cretaceous (Herrera-Flores *et al*., 2017, Fig. 1), even if the known fossil genera of similar age to the Burmese amber forest are all American rather than Asian. Determination of the host(s) of the fossil *A. kaufmani* sp. nov. would be an intriguing prospect, but for the moment remains equivocal.

### Tick evolutionary tree

**Fig. 9.**
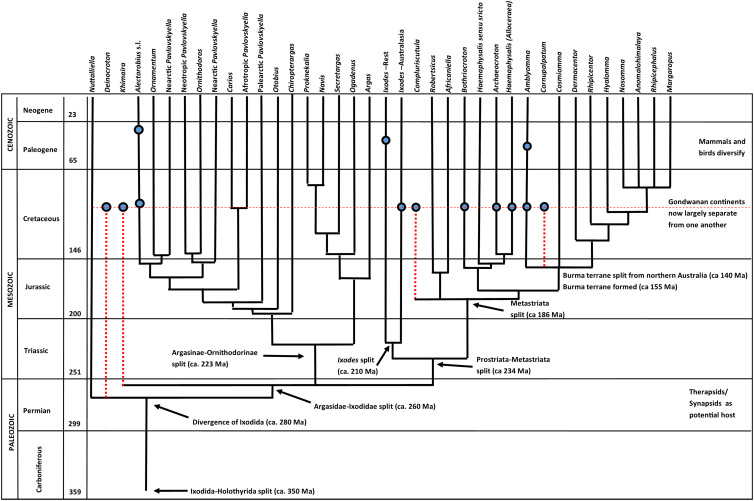
Evolutionary tree, revised and modified from Chitimia-Dobler *et al*. (2017, Fig. 3). Included here is Peñalver *et al*.'s (2017) extinct, mid-Cretaceous Burmese amber genus *Deinocroton*, which is thought to be close to *Nuttalliella*, the extinct genus *Khimaira* and the extant genera *Ixodes* and *Haemaphysalis* (Chitimia-Dobler *et al*., 2018, 2022), together with the new Cretaceous records of *Archaecroton* and *Bothriocroton* (this study). The phylogeny and estimated times of divergence are according to Barker and Murrell (2004), Mans *et al*. (2019), Mans *et al*. (2021) and Kelava *et al*. (2021).

As noted above, *Bothriocroton* and *Archaeocroton* belong to the Metastriata; a clade encompassing all hard ticks excluding *Ixodes*. In detail, Metastriata includes 14 extant and 2 extinct genera. Recent molecular studies have clarified relationships between metastriate genera to some extent (Mans *et al*., 2019, 2021; Kelava *et al*., 2021). As such, deep divergences exist at the base of the metastriate tree that result in various separate lineages (Fig. 9). This includes a separate lineage composed of *Archaeocroton*, *Bothriocroton*, *Haemaphysalis sensu stricto* and *Haemaphysalis* (*Allocereae*) and a lineage composed of *Africaniella* and *Robertsicus*. The extinct *Compluriscutula* may represent another distinct lineage closely related to either of these prior lineages. Another lineage includes *Amblyomma* and the Rhipicephalinae (*Anomalohimalaya*, *Cosmiomma*, *Dermacentor*, *Hyalomma*, *Margaropus*, *Rhipicephalus* and *Rhipicentor*). This latter lineage probably also includes the extinct *Cornupalpatum*. Possible reasons for the current inability to define the basal relationships of the metastriate genera more clearly may be the rapid diversification of lineages as well as possible extinction of various metastriate genera (represented by *Compluriscutula* and *Cornupalpatum*, but probably others as well). Previous hard ticks from Burmese amber documented Amblyomminae (Chitimia-Dobler *et al*., 2017) and Haemaphysalinae (Chitimia-Dobler *et al*., 2018) and now a third subfamily: Bothriocrotoninae. In the phylogeny presented above this represents one of the first lineages to branch off from the metastriate stem.

Metastriata is estimated to have split off from Prostriata (=*Ixodes*) in the Early Triassic (ca. 234 ± 18 Ma) with a further putative divergence into the modern genera in the Early Cretaceous at ca. 180 ± 15 Ma (Mans *et al*., 2019). In other words, the Burmese amber fossils assigned to the Amblyomminae, Haemaphysalinae and now also Bothriocrotoninae and Archaeocrotininae came from a time after the suggested radiation into living genera (Fig. 9). *Bothriocroton* and *Amblyomma*, representing the 2 most basal subfamilies (as well as *Archaeocroton*) tend to be found on reptiles (see also above), while *Haemaphysalis* tends to be associated with mammals. The subfamily Rhipicephalinae – which are exclusively found on placental mammals – may well be much earlier to the suggested radiation and is currently only known convincingly from a handful of subfossil records.

In this context, a putative Baltic amber example of the rhipicephaline genus *Hyalomma* in de la Fuente (2003) was treated as a misidentified caeculid mite by E. Sidorchuk in Chitimia-Dobler *et al*. (2017). Its interpretation as a tick was defended by Estrada-Peña and de la Fuente (2018), who suggested that the distinctive inward-facing ‘rakes’ on the legs could be artefacts of setae covered with debris. This interpretation remains unconvincing. The fossil in question was originally interpreted as male, and is now considered a female, and a clear expression of characters belonging to *Hyalomma*, or to ticks in general, is absent. For this reason, the amber *Hyalomma* record is excluded from Fig. 9 and suggests that it remains an unreliable calibration point for molecular clock studies as was used previously (Sands *et al*., 2017; Mans *et al*., 2019).

In conclusion, the Burmese amber fossils have to date yielded a remarkable assemblage of tick fossils of both extant and extinct families and genera. This includes the extinct families and genera Deinocrotonidae, Khimairidae, *Compluriscutula* and *Cornupalpatum*, respectively. It also includes the extant genera, notably the prostriate *Ixodes* and the metastriate *Amblyomma*, *Archaecroton*, *Bothriocroton* and *Haemaphysalis*, giving them minimum dates of origin of at least 100 Ma that serve as valuable calibration points for molecular dating. There is a ray of hope that future findings will highlight more genera that will deepen our understanding of tick evolution.

## Data Availability

All data generated or analysed during this study are included in this published article (and its supplementary files).

## Supplementary material

For supplementary material accompanying this paper visit https://doi.org/10.1017/S0031182022001585. click here to view supplementary material
