# Supplementary material for: Hard ticks in Burmese amber with Australasian affinities
Source: Parasitology. 2022 Nov 7;150(2):157–71. doi: 10.1017/S0031182022001585 (PMC10090639; doi:10.1017/S0031182022001585)
Supplement: Supplementary file 1 [file S0031182022001585sup001.docx]

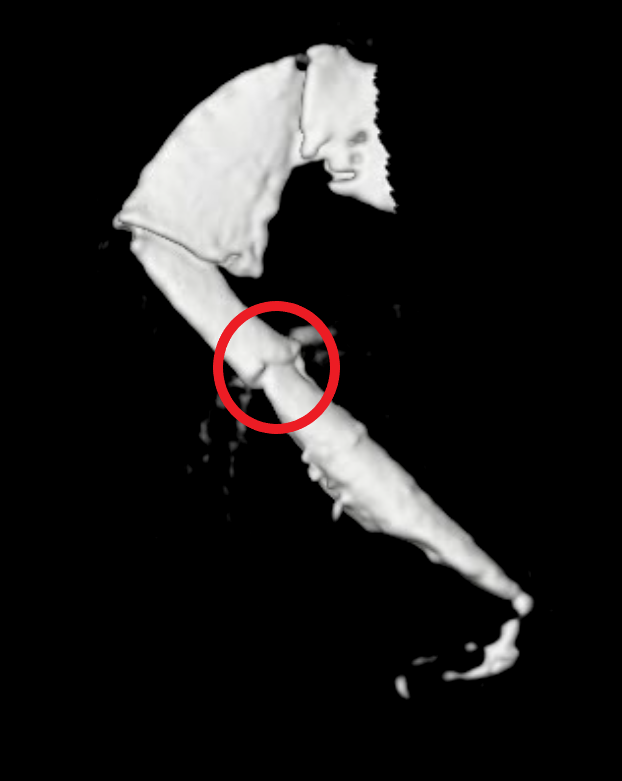


**Supplementary figure 1:** The pseudo-articulation found on tarsi 2 at one third of its length of *Archaecroton kaufmani* sp. nov. is indicated with a circle.
